# Automated synthesis and quality control of [^99m^Tc]Tc-PSMA for radioguided surgery (in a [^68^Ga]Ga-PSMA workflow)

**DOI:** 10.1186/s41181-020-00095-9

**Published:** 2020-05-01

**Authors:** Else A. Aalbersberg, Lotte van Andel, Martine M. Geluk-Jonker, Jos H. Beijnen, Marcel P. M. Stokkel, Jeroen J. M. A. Hendrikx

**Affiliations:** 1 Department of Nuclear Medicine, Netherlands Cancer Institute, Plesmanlaan 121, 1066 CX Amsterdam, the Netherlands; 2 Department of Pharmacy & Pharmacology, Netherlands Cancer Institute, Amsterdam, the Netherlands

**Keywords:** ^99m^Tc, PSMA, [^99m^Tc]Tc-PSMA, PSMA-I&S, Prostate cancer, Automated synthesis, Quality control, Radioguided surgery

## Abstract

**Background:**

Lymph node dissection is a therapeutic option for prostate cancer patients with a high risk of- or proven lymph node metastases. Radioguided surgery after intravenous injection of [^99m^Tc]Tc-PSMA could improve the selectivity of lymph node dissection. The aim of this project was to develop an automated synthesis method for [^99m^Tc]Tc-PSMA, using the disposables and chemicals used at our institute for [^68^Ga]Ga-PSMA labeling. Furthermore, quality control procedures and validation results of the automated production of [^99m^Tc]Tc-PSMA conform cGMP and cGRPP are presented.

**Methods:**

[^99m^Tc]Tc-PSMA is produced fully automatic with a Scintomics synthesis module. Quality control procedures are described and performed for: activity, labeling yield, visual inspection, pH measurement, sterility and endotoxin determination, radionuclide purity, radiochemical purity (^99m^Tc-colloids, unbound [^99m^Tc]pertechnetate, and other impurities), and HEPES content. Three batches of [^99m^Tc]Tc-PSMA were prepared on three separate days for validation and stability testing at 0, 4, 6, and 24 h.

**Results:**

[^99m^Tc]Tc-PSMA can be successfully manufactured automatically within a [^68^Ga]Ga-PSMA workflow with the addition of only [^99m^Tc]pertechnetate and stannous chloride. The radiochemical purity after production was highly reproducible (96.3%, 97.6%, and 98.2%) and remained > 90% (required for patient administration) up to 6 h later.

**Conclusion:**

A fully automated labeling procedure with corresponding quality control methods for production of [^99m^Tc]Tc-PSMA is presented, which is validated according to cGMP and cGRPP guidelines and can be implemented in a GMP environment. The produced [^99m^Tc]Tc-PSMA is stable for up to 6 h. The presented procedure is almost identical to the automated production of [^68^Ga]Ga-PSMA and can therefore be implemented expediently if a workflow for [^68^Ga]Ga-PSMA is already in place.

## Background

Prostate cancer is the second most commonly diagnosed cancer in males worldwide, and causes 6.7% of all cancer deaths in this group (Bray et al. 2018). In recent years, nuclear medicine imaging and therapy of prostate cancer radically changed through the introduction of radiolabeled PSMA (prostate specific membrane antigen) binding peptides. PSMA is a membrane glycoprotein that is upregulated in prostate cancer tissue, whilst expression is low in normal tissues surrounding the prostate (Sweat et al. 1998; Mannweiler et al. 2009). Radiolabeling of PSMA binding analogues with either Gallium-68 (^68^Ga) or Fluorine-18 (^18^F) allows for PET imaging for prostate cancer with high accuracy (Perera et al. 2016).

Diagnostic radiolabeled PSMA peptides are not only of interest for PET imaging, but also for radioguided surgery. When prostate cancer metastasizes, regional lymph nodes in the pelvic area (below the common iliac artery bifurcation) are the first to be affected. In this cases, lymph node dissection is the therapeutic option of choice. However, the extent of pelvic lymph node dissection is under scrutiny; increasing the extent of lymph node dissection increases complications, whilst the therapeutic effect in the long-term remains unknown (Ploussard et al. 2019; Fossati et al. 2017). Radioguided surgery could aid in the identification of cancer positive lymph nodes in real time. Making use of a gamma probe, surgeons are able to locate and to remove [^99m^Tc]Tc-PSMA positive lymph nodes which might lead to more selective lymph node dissections as compared to complete or extensive lymph node dissections. Several cases and studies of successful radioguided surgery with [^99m^Tc]Tc-PSMA have been described previously (Kratzik et al. 2018; Robu et al. 2017; Maurer et al. 2019). Furthermore, [^99m^Tc]Tc-PSMA SPECT/CT could be used in prostate cancer diagnostics in hospitals where PET/CT is not available.

Recently PSMA-I&S (PSMA-Imaging&Surgery), suitable for radiolabeling with ^99m^Tc, has become available commercially in GMP quality, whilst the kit formulation including the PSMA peptide and all excipients for labeling is not available in GMP grade. The synthesis of [^99m^Tc]Tc- PSMA has been described previously, but the method presented is a manual procedure (Robu et al. 2017). For implementation of [^99m^Tc]Tc-PSMA at our hospital, automated synthesis was preferred in order to reduce the radiation dose to personnel and to standardize the process. In addition, the use of disposables already available without the introduction of new disposables or chemicals was preferred as this increases the speed for implementation of novel radiopharmaceuticals, reduces the number of items required in stock, and avoids the need for qualification of new suppliers conform the current principles of Good Manufacturing Practice (cGMP) and Good Radiopharmacy Practice (cGRPP) (Elsinga et al. 2010).

The aim of this study is to present an automated synthesis method for [^99m^Tc]Tc-PSMA, using the disposables and chemicals used at our institute for [^68^Ga]Ga-PSMA labeling. Furthermore, quality control procedures and validation results of the automated production of [^99m^Tc]Tc-PSMA conform cGMP are presented.

## Methods

### General

#### Consumables

GMP grade PSMA-I&S in 40 μg vials (2-mercaptoacetyl-D-Ser-D-Ser-D-Ser--D-Tyr-D-2-Nal-D-Lys (SUB-L-Lys-Urea-L-Glu)) was obtained from piCHEM (Raaba-Grambach, Austria). GMP grade Stanno-chloride 1 mg/mL was purchased from Apotheek Martini Ziekenhuis (Groningen, the Netherlands) in single use ampoules of 2 ml. The reagent and hardware kit (cassette) for synthesis of ^68^Ga peptides designed for Scintomics GRP synthesizer was purchased from ABX (Radeberg, Germany). [^99m^Tc]pertechnetate was obtained from GE Healthcare Radiopharmacy (Leiderdorp, the Netherlands). For the eluent solutions, acetonitrile HPLC-S gradient grade was obtained from Biosolve BV (Valkenswaard, the Netherlands), trifluoroacetic acid ≥99.8% (TFA) from Merck Millipore (Darmstadt, Germany) and sterile water for irrigation from B Braun (Melsungen, Germany).

#### Automated radiolabeling

**Fig. 1:**
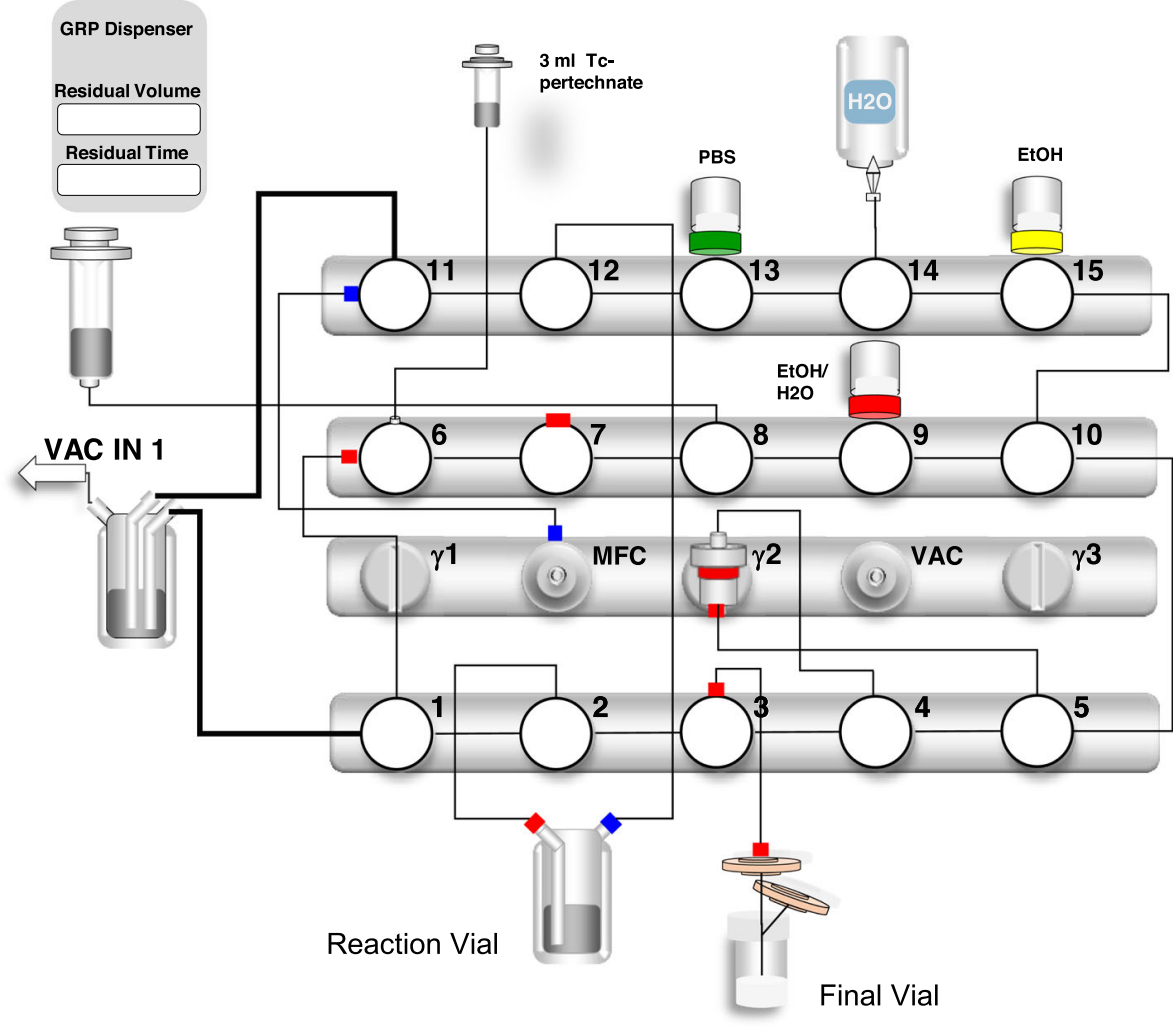
Set-up of the automated Scintomics system for radiolabeling of ^99m^Tc-PSMA

Automated labeling was performed with a Scintomics GRP 3 V module (Scintomics GmbH, Fürstenfeldbruck, Germany). The cassette was assembled according to the manufacturer’s instructions for production of ^68^Ga-peptides with three exceptions: (1) the gallium generator was not attached to the system and this outlet was capped, (2) the syringe with 5 M NaCl was discarded and replaced with a shielded syringe with 3 mL [^99m^Tc] pertechnetate (2000 MBq), and (3) the PS-H^+^ cartridge was discarded since the [^99m^Tc]pertechnetate does not need to be purified further. PSMA was dissolved in 1.5 mL of 1.5 M HEPES buffer and added to the reaction vial, supplemented with 20 μL of 1 mg/mL stannous chloride. Figure 1 shows a schematic drawing of the system used.

**Fig. 2:**
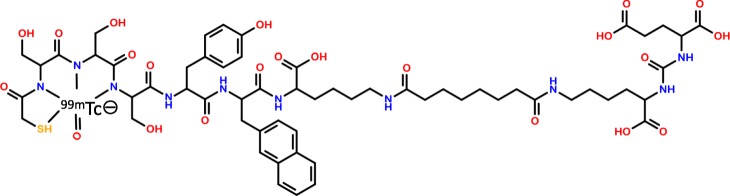
Structure of [^99m^Tc]Tc-PSMA-I&S

The labeling process consists of the following steps: (1) Conditioning of the Sep-Pak Light C18-cartrigde with ethanol absolute, (2) rinsing of the C18-cartrigde with water, (3) addition of [^99m^Tc]pertechnetate to the reaction vial containing the dissolved peptide, (4) heating of the reaction vial to 100 °C for 20 min, (5) cooling to room temperature, (6) transfer of the labeled peptide to the C18-cartrigde, (7) rinsing of the C18-cartrigde with water, (8) elution of the labeled peptide from the C18-cartrigde with ethanol:water (1:1 v/v), to the final vial (including a sterile filtration step with a 0.22 μm filter), and (9) dilution of the labeled peptide with phosphate buffered saline (pH 7.5) to a final volume of 14 mL. The final product is shown in Fig. 2.

#### Activity and yield

The activity of the produced [^99m^Tc]Tc-PSMA was measured in a dose calibrator (Comecer Netherlands, Joure, the Netherlands). The yield was calculated as follows: Yield (%) = Activity [^99m^Tc]Tc-PSMA (MBq) / Activity [^99m^Tc]pertechnetate (MBq) * 100%, in which the activity of ^99m^Tc is corrected for decay to the activity reference time of [^99m^Tc]pertechnetate.

#### Visual inspection and pH

Visual inspection was performed; the final product should be colorless and clear of any visible particles. The pH was measured with a pH indicator strip (Merck Millipore, Darmstadt, Germany) and should lie between 7 and 8.

#### Sterility and endotoxins

Sterility was determined by adding 1 mL of ^99m^Tc-PSMA to 10 mL tryptic soy broth (TSB) (Biotrading, Mijdrecht, the Netherlands) and incubated for 7 days at 32.5 °C. The medium of the TSB should be clear after the incubation period. Endotoxins were determined according to the European Pharmacopoeia 10.0, chapter 2.6.14 bacterial endotoxins with method A (Gel-clot method: limit test) (European Pharmacopoeia 10.0 n.d.-a). The limit of endotoxins is < 50 IU/2 mL, based on the limit of 2.5 IU/kg for radiopharmaceuticals (European Pharmacopoeia 10.0 n.d.-b), with an average patient of 70 kg and an a maximum injection volume of 7 mL.

#### Radionuclide identity

[^99m^Tc]Pertechnetate (Ph Eur quality) in a 3 mL syringe was ordered from a central pharmacy. Therefore, the identity of ^99m^Tc was verified according to the Ph Eur monograph of [^99m^Tc]pertechnetate by placing 1 mL of a 500x diluted ^99m^Tc-PSMA sample in a well counter (Canberra, Mirion Technologies, San Ramon, United States) and acquiring a spectrum for 30s. The radionuclide identity was accepted if the most prominent gamma photon had an energy of 141 keV (European Pharmacopoeia 10.0 n.d.-c).

#### iTLC for determination of ^99m^Tc-colloids

Since ^99m^Tc-colloids cannot be quantified with HPLC, instant thin layer chromatography (iTLC) was used to determine the fraction of ^99m^Tc-colloids in the final product. The solid phase consisted of glass microfiber chromatography paper impregnated with silica gel (Agilent, Santa Clara, USA) with a mobile phase of ammonium acetate (1 M in water):methanol 50:50 v/v. All samples were diluted 1:25 in water and 5 μL was applied on the iTLC-SG paper. When the solvent front had traveled at least 10 cm, the chromatography paper was cut horizontally into 1 cm pieces and the total radioactivity in each piece was determined with a well counter with multichannel analyzer (Canberra, Mirion Technologies, San Ramon, USA). The procedure was performed in duplicate for each measurement. The retention factor (RF) of ^99m^Tc-colloids is ≤0.2 and the RF of [^99m^Tc]Tc-PSMA ≥0.7. The percentage of colloids was determined as follows: % ^99m^Tc-colloids = counts RF ≤ 0.2 / (counts RF ≤ 0.2 + counts RF ≥ 0.7) * 100. Up to 10% of ^99m^Tc-colloids are accepted in the final product.

#### HPLC for determination of unbound[^99m^Tc]pertechnetate and other impurities

High performance liquid chromatography (HPLC) was performed with a Dionex ultimate 3000 UHPLC system coupled to a Dionex 3000 UV detector (ThermoFisher Scientific, Waltham, USA) and a Canberra Packard flow scintillation analyzer with a gamma cell (Canberra Packard GmbH, Schwadorf, Austria). Eluent A contained 1% TFA in water and eluent B contained 1% TFA in acetonitrile. Samples were diluted 10x in water prior to injection and analyzed with a reversed phase C18 column (4.6 × 250 mm, 5 μm) (Waters Symmetry Shield) and a linear gradient of 85% A and 15% B to 65% A and 35% B in 18 min followed by 2 min of 85% A with a flow rate of 1.5 ml/min. Radiation detection was done between 0 and 300 keV. Unbound [^99m^Tc]pertechnetate had a retention time of 2.5 min. The percentage of [^99m^Tc]pertechnetate and other impurities were calculated based on the area of the respective peaks compared the total area of all peaks. Impurities were defined as peaks within the first 10 min larger than 1%. The accepted value of [^99m^Tc]pertechnetate in the final product is ≤2%.

#### Radiochemical purity

The radiochemical purity (defined as percentage [^99m^Tc]Tc-PSMA) was calculated according to the following formula: radiochemical purity = 100% – % ions determined with HPLC – % impurities detected with HPLC – % colloids measured with iTLC. For acceptance of the final product, the radiochemical purity should be ≥90% (European Pharmacopoeia 10.0 n.d.-d).

#### iTLC for determination of HEPES concentration

The HEPES concentration in the final product was determined after decay of the radioisotope according to the test for HEPES in the European Pharmacopoeia monograph of ^68^Gallium edotreotide injection (European Pharmacopoeia 10.0 n.d.-e). As a reference solution, HEPES (Sigma Aldrich, St. Louis, United States) was dissolved in water with a concentration of 40 μg/ml. 3 μL of reference solution or [^99m^Tc]Tc-PSMA solution were brought onto a ITLC-SG F_254_ plate (Merck, Darmstadt, Germany) and placed in a mobile phase containing water:acetonitrile 25:75 v/v. When the solvent front had traveled over at least 2/3 of the plate, the plate was placed in an iTLC chamber containing iodine crystals (Sigma Aldrich, St. Louis, United States) for at least 4 min. The yellow spot of the [^99m^Tc]Tc-PSMA solution should not be more intense than that of the reference solution (< 200 μg/patient dosage).

### Development of the labeling

#### Stannous chloride addition

[^99m^Tc]Pertechnetate needs to be reduced to a lower oxidation state in order to form complexes coupled to ^99m^Tc. This is achieved through the addition of a reducing agent. Stannous chloride is the reducing agent used in ^99m^Tc preparations. In commercially available cold kits, 0.0076–0.5 mg of SnCl_2_x2H_2_O is added, leading to a Sn/Tc ratio of 2 × 10^3^–1.2 × 10^5^ (Spies and Pietzsch 2007). Therefore, we added 40, 200, or 500 μg of SnCl_2_x2H_2_O to the dissolved PSMA prior to radiolabeling with 2000 MBq [^99m^Tc]pertechnetate (*n* = 1 each, Sn/Tc ratio of 1.7 × 10^3^, 8.5 × 10^3^ and 2.1 × 10^4^ respectively, calculated with a theoretical specific activity for 99mTc of 1.95 × 1017 Bq/g).

#### iTLC development

To obtain adequate separation between ^99m^Tc-colloids and [^99m^Tc]Tc-PSMA, both the mobile phase and the length of the stationary phase were investigated. Three mobile phases were tested: 0.9% NaCl in water, water/acetonitrile 40:60 v/v, and 1 M ammonium acetate/methanol 50:50 v/v. Furthermore, the length of the stationary phase with either 5 or 10 cm was tested. The colloid [^99m^Tc]Tc-nanocoll was used as a surrogate for ^99m^Tc-colloids to be a positive control in the validation.

### Validation and stability

Three manufacturing processes of [^99m^Tc]Tc-PSMA were executed on three separate days. All quality control measurements were carried out immediately after production. For stability testing, the product was stored in the final vial and in a prepared syringe for 4, 6 and 24 h, both kept at room temperature in lead shielding. At each time point, all quality control measurements were performed, except testing for sterility, endotoxins, radionuclide purity and HEPES concentration.

## Results

### Development of the labeling

#### Stannous chloride addition

40, 200, or 500 μg of SnCl_2_x2H_2_O was used as reducing agent prior to labeling. In all three conditions, the remaining amount of unbound [^99m^Tc]pertechnetate was very low: 0.10% with 40 μg, 0.08% with 200 μg, and 0.09% with 500 μg SnCl_2_x2H_2_O. Therefore, the lowest amount of of SnCl_2_x2H_2_O (40 μg) was chosen for validation as this was sufficient for reduction of [^99m^Tc]pertechnetate.

#### iTLC development

**Fig. 3:**
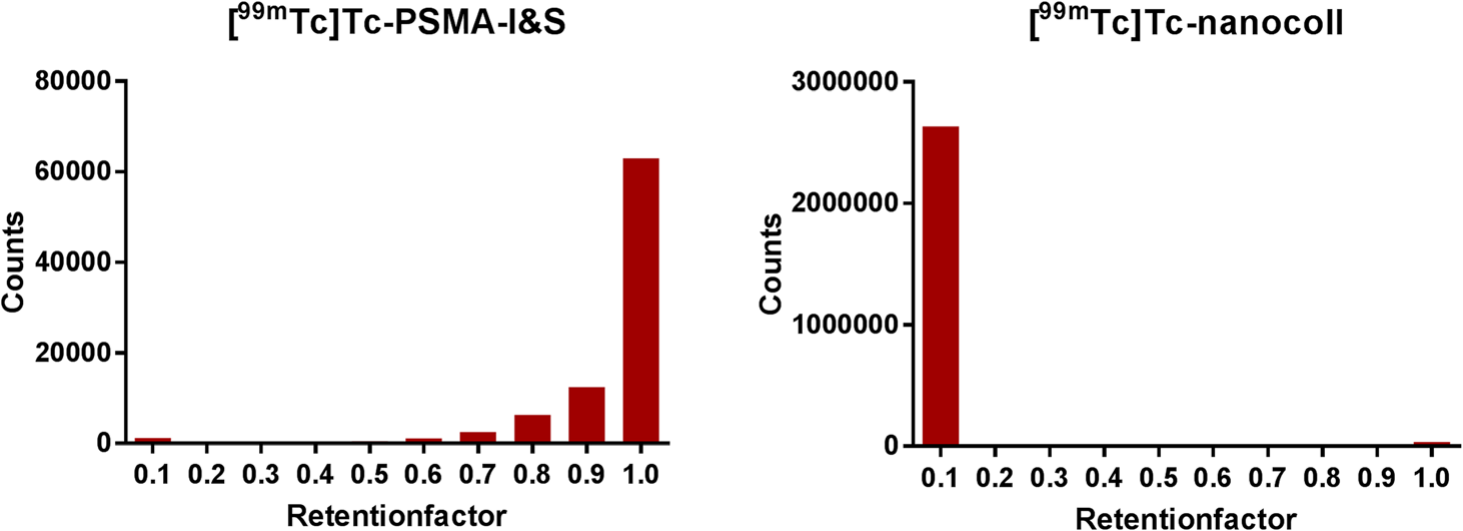
Instant thin layer chromatography of both [^99m^Tc]Tc-nanocoll and [^99m^Tc]Tc-PSMA. The retention factor of ^99m^Tc-colloids is less than 0.2 and that of [^99m^Tc]Tc-PSMA greater than 0.7

1 M ammonium acetate/methanol 50:50 v/v over a 10 cm stationary phase led to the best separation of ^99m^Tc-colloids and [^99m^Tc]Tc-PSMA and is shown in Fig. 3.

### Validation of the labeling

**Fig. 4:**
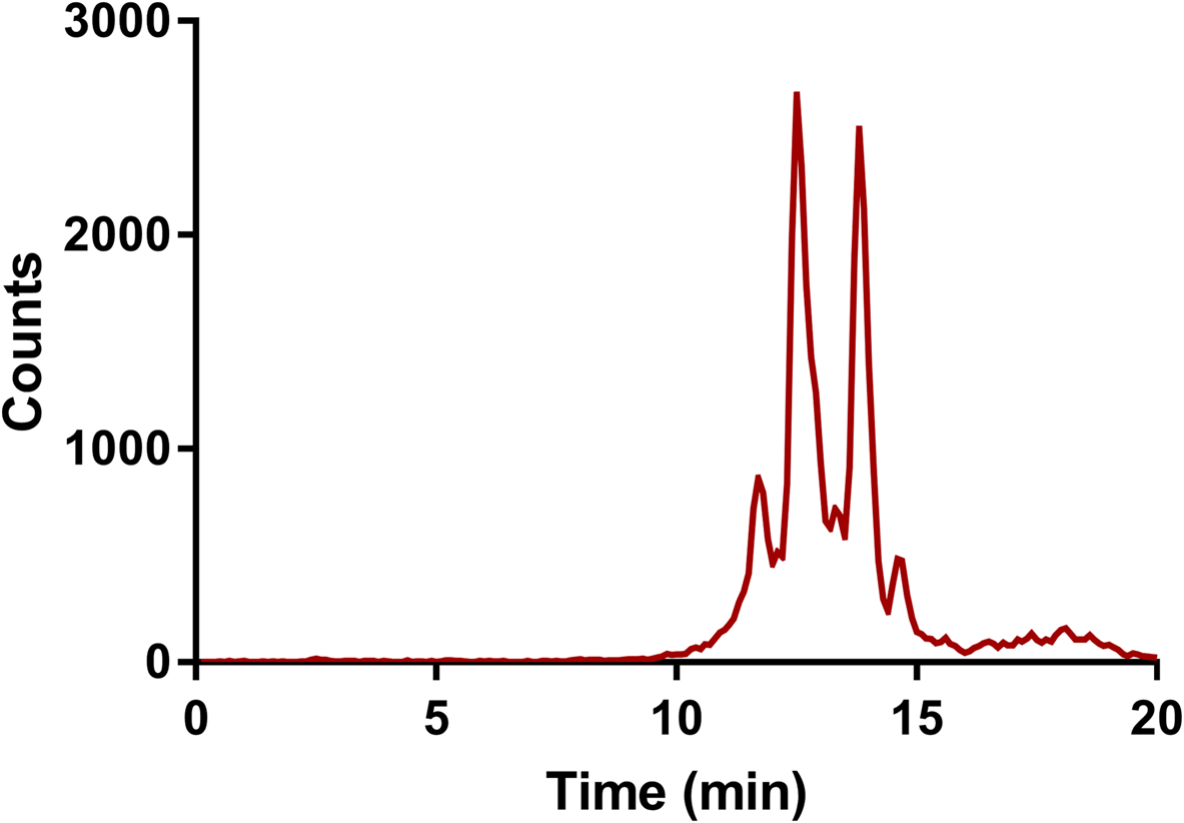
High performance liquid chromatography (HPLC) chromatogram of the radiodetector. The retention time of unbound [^99m^Tc]pertechnetate is 2.5 min. The retention time of [^99m^Tc]Tc-PSMA is between 10 and 15 min, with different peaks accounting for different protonation states

Three automatic productions of [^99m^Tc]Tc-PSMA were successfully completed. All results are summarized in Table 1. Preparation and set-up of the labeling system and consumables took ~ 30 min, the automated labeling was performed in ~ 45 min and immediate quality control procedures (visual inspection, pH, ^99m^Tc-colloids, ^99m^Tc-ions, and radiochemical purity) can be performed in < 30 min. The mean yield of produced [^99m^Tc]Tc-PSMA was 75.9% of the initial activity at start of the labeling (corrected for decay, range 74.8–77.9%), allowing for multiple patient administrations from one batch, depending on the chosen administered activity, which ranges from 200 to 850 MBq in literature (Kratzik et al. 2018; Robu et al. 2017; Maurer et al. 2019). Figure 4 shows a representative HPLC chromatogram for [^99m^Tc]Tc-PSMA.

**Table 1:** Results of three validation batches of [^99m^Tc]Tc-PSMA

Item	Time (hours)	Batch 1		Batch 2		Batch 3	
		*Syringe*	*Vial*	*Syringe*	*Vial*	*Syringe*	*Vial*
**Yield** *(Corrected for decay)*	*T = 0*	75.05%		77.94%		74.82%	
**Visual inspection***(Criteria: colorless without visible particles)*	*T = 0*	Colorless No particles		Colorless No particles		Colorless No particles	
	*T = 4*	Colorless No particles	Colorless No particles	Colorless No particles	Colorless No particles	Colorless No particles	Colorless No particles
	*T = 6*	Colorless No particles	Colorless No particles	Colorless No particles	Colorless No particles	Colorless No particles	Colorless No particles
	*T = 24*	Colorless No particles	Colorless No particles				
**pH** *(Criteria: pH 4,0-8,0)*	*T = 0*	7.5		7.5		7.5	
	*T = 4*	7.5	7.5	7.5	7.5	7.5	7.5
	*T = 6*	7.5	7.5	7.5	7.5	7.5	7.5
	*T = 24*	7.5	7.5				
**Sterility** *(Criteria: TSB clear after 7-day incubation)*	*T = 0*	TSB clear		TSB clear		TSB clear	
**Endotoxin** *(Criteria: < 50 IU / 2 mL)*	*T = 0*	< 50 IU/2 ml		< 50 IU/2 ml		< 50 IU/2 ml	
**Radionuclide purity** ***(****Main peak at 141 keV)*	*T = 0*	Main peak at 141 keV		Main peak at 141 keV		Main peak at 141 keV	
**Tc-99m colloids** *(Criteria: ≤10%)*	*T = 0*	1,46%		1,92%		2,82%	
	*T = 4*	6,56%	7,02%	6,83%	7,39%	7,81%	6,64%
	*T = 6*	6,65%	7,18%	7,60%	6,47%	8,51%	8,40%
	*T = 24*	10,96% *	9,44%				
**Tc-99m ions** (*Criteria: ≤2%)*	*T = 0*	0,34%		0,48%		0,84%	
	*T = 4*	0,68%	1,06%	0,28%	0,78%	1,22%	0,90%
	*T = 6*	0,58%	1,02%	0,82%	1,10%	1,10%	1,56%
	*T = 24*	1,46%	1,34%				
**Radiochemical purity** *(Criteria: ≥90%)*	*T = 0*	98,20%		97,60%		96,34%	
	*T = 4*	92,76%	91,92%	92,89%	91,83%	90,97%	92,46%
	*T = 6*	92,77%	91,80%	91,58%	92,43%	90,39%	90,04%
	*T = 24*	87.58% *	89,22% *				
**HEPES** (Criteria: < 200 μg/patient dosage)	*T = 0*	< 200 μg/patient dose		< 200 μg/patient dose		< 200 μg/patient dose	

The results of the three validation batches (batch 1, batch 2, batch 3) are shown at different time points (t = 0, t = 2, t = 6, and t = 24 h). At t = 0 one sample was taken, whilst at the other time points a samples was measured from either the final vial or a prepared patient syringe kept at room temperature. The accepted limit/criteria is noted in the first column. * indicates that the value does not meet the criteria

The stability of [^99m^Tc]Tc-PSMA was evaluated 4, 6 and 24 h after manufacturing. At later time-points the amount of ^99m^Tc-colloids in the final product increased, leading to a decrease in radiochemical purity. The percentage of unbound [^99m^Tc]pertechnetate remained fairly stable over time and no other impurities were formed. The stability of the batches was acceptable up to 6 h after production, whilst after 24 h the radiochemical purity of the first batch was below the accepted limits, and this time point was therefore not measured in subsequent validation batches. There is no difference in stability of the [^99m^Tc]-PSMA when stored in the final vial or in a prepared patient syringe.

## Discussion

Prostate cancer is a common disease among older men. Lymph node dissection is a therapeutic option for patients with both either primary prostate cancer and a high risk of lymph node metastases or in patients with a biochemical recurrence with lymph node metastases (Ploussard et al. 2019; Fossati et al. 2017). Since extensive lymph node dissections have a risk of both perioperative and postoperative complications, selective lymph node dissection is preferred. Radioguided surgery with a gamma probe after intravenous injection of [^99m^Tc]Tc-PSMA could lead to selective lymph node dissection of cancer positive lymph nodes only.

Previously, a kit preparation for [^99m^Tc]Tc-PSMA has been developed (Robu et al. 2017) . However, this kit is not approved by the European Medicines Agency (EMA). Due to regulatory issues in several countries including the Netherlands, these unregistered kits cannot be used universally. Therefore, this automated production with a commercially available (GMP produced) PSMA precursor has been developed.

This research describes the automatic production and subsequent quality control methods of [^99m^Tc]Tc-PSMA. Automated standardized production of radiopharmaceuticals has two main advantages; (1) the technical precision and reproducibility between productions is high, and (2) the radiation exposure of personnel is kept to a minimum, which is essential in a high-throughput department where multiple (different) radiopharmaceuticals are produced each day. Previously described procedures in literature all consisted of manual production methods, which can performed in a short period of time (Table 2) (Kratzik et al. 2018; Robu et al. 2017). Although our automated method might slightly increase the time needed for the production (1.25 h), personnel only needs to be present for 30 min for set-up of the automatic system. The half-life of [^99m^Tc]Tc-PSMA allows extensively for the time needed for set-up of the system and subsequent production and quality control.

**Table 2:** Comparison between the automated method described in this article and the methods described in literature

	Aalbersberg et al. (current method)	Robu et al. Single method	Robu et al. Kit method	Kratzik et al.
**Method**	Automated	Manual	Manual	Manual
**Buffer**	HEPES	Ammonium acetate & disodium tartrate	Sodium hydrogen phosphate & Sodium tartrate	Unknown
**Peptide**	40 μg PSMA-I&S (=28.7 nmol)	20–30 nmol PSMA-I&S	25 nmol PSMA-I&S	PSMA-I&S (unknown quantity)
**Temperature and time**	100 °C 20 min	90 °C 20 min	90 °C 20 min	100 °C 10 min
**C**^**−18**^**cartridge purification**	Yes	Yes	No	No

In order to ensure a fast and successful implementation of the production of ^99m^Tc-PSMA, the labeling procedure was almost identical to that of [^68^Ga]Ga-PSMA, with minor changes (^68^Ga-generator replaced by [^99m^Tc]pertechnetate syringe, and addition of stannous chloride to the dissolved peptide). This limits the number of new products to be introduced into the laboratory and minimizes the training time of staff members.

The stability of [^99m^Tc]Tc-PSMA produced by the described method is 6 h at room temperature. This offers ample opportunity for consecutive administration, imaging, and/or surgery of multiple patients. The [^99m^Tc]Tc-PSMA was not stable for 24 h. However, within this research, improvement of the stability up to 24 h was not investigated since this is not necessary at our institute. If extended stability is necessary, this should be investigated separately.

## Conclusion

A fully automated labeling procedure for production of [^99m^Tc]Tc-PSMA is presented, which is validated according to cGMP and cGRPP guidelines and is suitable for implementation in a cGMP environment. The produced [^99m^Tc]Tc-PSMA is stable for up to 6 h. The presented method is almost identical to the automated production of [^68^Ga]Ga-PSMA and can therefore be implemented expediently if a workflow for [^68^Ga]Ga-PSMA is already in place.

## Data Availability

The datasets used and/or analysed during the current study are available from the corresponding author on reasonable request.

## References

[CR1] Bray F, Ferlay J, Soerjomataram I, Siegel RL, Torre LA, Jemal A (2018). Global cancer statistics 2018: GLOBOCAN estimates of incidence and mortality worldwide for 36 cancers in 185 countries. CA Cancer J Clin.

[CR2] Elsinga P, Todde S, Penuelas I (2010). Guidence on current good radiopharmacy practice (cGRPP) for the small-scale preparation of radiopharmaceuticals. Eur J Nucl Med Mol Imaging.

[CR3] European Pharmacopoeia 10.0. (n.d.-a) Paragraph 2.6.14 Bacterial endotoxins. 01/2018:20614; 2020.

[CR4] European Pharmacopoeia 10.0. (n.d.-b) Paragraph 5.1.10 Guidelines for using the test for bacterial endotoxins. 07/2016:50110.

[CR5] European Pharmacopoeia 10.0. (n.d.-c) Monograph Sodium pertechnetate (^99m^Tc) injection (non-fission). 01/2008:0283. Corrected 7.0.

[CR6] European Pharmacopoeia 10.0. (n.d.-d) Paragraph 2.9.6 Uniformity of content of single-dose preparations. 01/2017:20906.

[CR7] European Pharmacopoeia 10.0. (n.d.-e) Monograph Gallium (68Ga) edotreotide injection. 01/2013:2482. Corrected 9.6.

[CR8] Fossati N, Willemse PPM, Van den Broeck T (2017). The benefits and harms of different extents of lymph node dissection during radical prostatectomy for prostate Cancer: a systematic review. Eur Urol.

[CR9] Kratzik C, Dorudi S, Schatzl M, Sinzinger H (2018). Tc-99m-PSMA imaging allows successful radioguided surgery in recurrent prostate cancer. Hell J Nucl Med.

[CR10] Mannweiler S, Amersdorfer P, Trajanoski S, Terrett JA, King D, Mehes G (2009). Heterogeneity of prostate-specific membrane antigen (PSMA) expression in prostate carcinoma with distant metastasis. Pathol Oncol Res.

[CR11] Maurer T, Robu S, Schottelius M (2019). 99m technetium-based prostate-specific membrane antigen–radioguided surgery in recurrent prostate Cancer. Eur Urol.

[CR12] Perera M, Papa N, Christidis D (2016). Sensitivity, specificity, and predictors of positive 68Ga-prostate-specific membrane antigen positron emission tomography in advanced prostate Cancer: a systematic review and meta-analysis. Eur Urol.

[CR13] Ploussard G, Gandaglia G, Borgmann H (2019). Salvage lymph node dissection for nodal recurrent prostate Cancer: a systematic review. Eur Urol.

[CR14] Robu S, Schottelius M, Eiber M (2017). Preclinical evaluation and first patient application of 99mTc-PSMA-I&S for SPECT imaging and radioguided surgery in prostate cancer. J Nucl Med.

[CR15] Spies H, Pietzsch H-J, Zolle I (2007). Stannous chloride in the preparation of 99mTc pharmaceuticals. Technetium-99m pharmaceuticals, preparation and quality control in nuclear medicine.

[CR16] Sweat SD, Pacelli A, Murphy GP, Bostwick DG (1998). Prostate-specific membrane antigen expression is greatest in prostate adenocarcinoma and lymph node metastases. Urology.

